# EPEC autotransporter adhesin (Eaa): a novel adhesin identified in atypical enteropathogenic *Escherichia coli*

**DOI:** 10.3389/fcimb.2025.1617101

**Published:** 2025-08-18

**Authors:** Henrique Orsi, Daiany R. P. de Lira, Ivana G. Castilho, Guilherme F. R. de Souza, Tugce Onur, Rosa M. Chura-Chambi, Cecilia M. Abe, Enéas Carvalho, Luis F. dos Santos, David A. Rasko, Mark A. Schembri, Angela S. Barbosa, Waldir P. Elias, Rodrigo T. Hernandes

**Affiliations:** ^1^ Universidade Estadual Paulista (UNESP), Instituto de Biociências, Botucatu, Brazil; ^2^ Laboratório de Bacteriologia, Instituto Butantan, São Paulo, Brazil; ^3^ School of Chemistry and Molecular Biosciences, The University of Queensland, Brisbane, QLD, Australia; ^4^ Centro de Bacteriologia, Instituto Adolfo Lutz, São Paulo, Brazil; ^5^ Department of Microbiology and Immunology, University of Maryland School of Medicine, Baltimore, MD, United States; ^6^ Institute for Genome Sciences, University of Maryland School of Medicine, Baltimore, MD, United States; ^7^ Institute for Molecular Bioscience (IMB), The University of Queensland, Brisbane, QLD, Australia; ^8^ Australian Infectious Diseases Research Centre, The University of Queensland, Brisbane, QLD, Australia

**Keywords:** autotransporter protein, adhesin, atypical EPEC, bacterial pathogenesis, biofilm, fibronectin

## Abstract

Enteropathogenic *Escherichia coli* (EPEC) is a pathogen that causes diarrhea that can be subdivided into typical (tEPEC) and atypical (aEPEC), based on the production of an adhesin termed Bundle-Forming Pilus (BFP) in the former group. aEPEC is one of the main bacterial pathogens isolated from individuals with diarrhea, and some serotypes have been implicated in diarrheal outbreaks in Brazil, such as the O2:H16. A comparative genomic analysis of aEPEC of this serotype led to the identification of a gene encoding a previously uncharacterized autotransporter protein. In the present study, this novel autotransporter protein was characterized and named EPEC Autotransporter Adhesin (Eaa). The Eaa-encoding gene (*eaa*) is located in a chromosomal prophage region of 17,014 base pairs, organized in 20 open reading frames and inserted downstream to the threonine-tRNA. A recombinant plasmid termed pIC (pBAD/*Myc*-His A harboring the *eaa* gene from aEPEC BA92) was transformed in the MS427 host bacteria, and the MS427(pIC) was used in phenotypic assays. Immunogold-labelling transmission electron microscopy, using anti-Eaa antibodies, showed the presence of Eaa in the cell surface of the wild-type BA92 and MS427(pIC) strains. Subsequently, we demonstrated that Eaa mediates bacterial autoaggregation, biofilm formation and binding to several components of the extracellular matrix, including fibrinogen, plasma and cellular fibronectin, type I, III as well as V collagen and laminin. In summary, we demonstrated that Eaa harbors several adherence properties and may contribute to the pathogenicity of some aEPEC isolates by mediating the interaction of this pathogen with biotic and abiotic surfaces.

## Introduction

Enteropathogenic *Escherichia coli* (EPEC) is one of the six pathotypes of diarrheagenic *E. coli* (DEC). The hallmark of EPEC infection is the attaching and effacing (AE) lesion formation on the surface of infected epithelial cells (Nataro and Kaper, 1998). The AE lesion is primarily characterized by the intimate adherence of the bacteria to the host epithelial cells, microvilli effacement and finally the formation of a pedestal-like structure rich in polymerized F-actin on the epithelial cell surface (Moon et al., 1983). The proteins necessary for the establishment of the AE lesion are encoded by ~40 genes located in a chromosomal pathogenicity island termed locus of enterocyte effacement, or LEE region (McDaniel et al., 1995). Among the genes in the LEE region are those that encode proteins that participate in the assembly of a Type 3 Secretion System (T3SS), the adhesin known as intimin, as well as the translocated intimin receptor named Tir, which is a bacterial protein that is translocated into the host cell by the T3SS (Jerse et al., 1990; Jarvis et al., 1995; Kenny et al., 1997).

EPEC can be divided into typical (tEPEC) and atypical (aEPEC) based on the presence of the genes for the production of a type IV fimbriae called bundle-forming pili (BFP) only in the former group (Trabulsi et al., 2002; Hernandes et al., 2009). The genes that encode the proteins that participate in the BFP biogenesis are located on a large plasmid called EPEC adherence factor (pEAF) (Girón et al., 1991; Donnenberg et al., 1992). BFP mediates the formation of compact bacterial microcolonies on the surface of infected cells and this pattern of adherence is referred to as localized adherence (LA) (Scalestky et al., 1984).

Since aEPEC does not produce BFP, these strains adhere to epithelial cells forming loose microcolonies on their surface, and this pattern of adherence is known as localized adherence-like (LAL) (Rodrigues et al., 1996). Although more sporadic, some aEPEC strains adhere to the host epithelial cells producing adherence patterns distinct from LAL, such as the aggregative or diffuse (Gomes et al., 1998, 2011; Abe et al., 2009; Vieira et al., 2019). Besides the *eae* gene, that encodes the adhesin intimin necessary for the establishment of the LAL pattern in aEPEC (Pelayo et al., 1999; Carvalho et al., 2005), many other adhesin-encoding genes have been detected in aEPEC strains, such as *paa*, *ecpA*, *lpfA*, *efa1*/*lifA* and *iha* (Gomes et al., 2011; Vieira et al., 2016; Munhoz et al., 2018).

aEPEC is one of the most frequently DEC pathotypes isolated from individuals with diarrhea in Brazil (Bueris et al., 2007; Araujo et al., 2007; Dias et al., 2016; Ori et al., 2018), as well as worldwide (Kotloff et al., 2013). Additionally, some aEPEC serotypes have been implicated as agents of diarrhea outbreaks (Hernandes et al., 2009). One example of this is the serotype O2:H16 isolates associated with a Brazilian outbreak of diarrhea (Vieira et al., 2016). In a previous comparative genetic study from our group, we compared 106 aEPEC from Brazil, including 7 strains of the serotype O2:H16, with 221 diverse global aEPEC (Hernandes et al., 2020). The genomic dataset generated in this study was used here to investigate a set of genes unique to aEPEC of serotype O2:H16 and led to the identification of a gene encoding a predicted uncharacterized autotransporter protein.

Autotransporters are a superfamily of proteins that use the type 5 secretion pathway for their delivery to the surface of Gram-negative bacteria, including EPEC (Abreu et al., 2013; Clarke et al., 2022; Henderson et al., 2004). The proteins of this superfamily possess a unifying structure comprising three functional domains: a signal peptide, responsible for the transport through the inner membrane via the Sec machinery; the passenger domain, which is the functional component of the protein, and a carboxy- terminal domain that forms a beta-barrel pore to allow the translocation of the passenger domain of the protein to the extracellular environment (Henderson et al., 2004; Leyton et al., 2012). These proteins can have a myriad of functions participating in several stages of the infectious process by mediating adherence to host epithelial cells and/or extracellular matrix (ECM) components, as well as invasion, biofilm formation, bacterial autoaggregation, serum resistance, or even acting as cytotoxins or proteases (Leyton et al., 2012; Meuskens et al., 2019).

Therefore, our main objective was to evaluate the ability of this novel autotransporter adhesin, termed here EPEC Autotransporter Adhesin (Eaa), to mediate bacterial autoaggregation, biofilm formation, as well as binding to various ECM components.

## Materials and methods

### Bacterial strains and plasmids used in this study

The aEPEC strain BA92 used in this study belongs to the serotype O2:H16 and was isolated during an epidemiological study carried out in the city Salvador, State of Bahia, Brazil (Bueris et al., 2007; Abe et al., 2009). All other bacterial strains and plasmids employed are listed in **Table 1**. Routinely, these strains were grown in lysogeny broth (LB) at 37°C, supplemented as appropriate with kanamycin (50 µg/mL) or ampicillin (100 µg/mL), and stored at -80°C in LB supplemented with 30% glycerol.

**Table 1:** Bacterial strains and plasmids used in this study.

Strains and plasmids	Relevant characteristic(s)	Reference
Strains		
BA92	Wild-type aEPEC isolate of serotype O2:H16	(Bueris et al., 2007; Abe et al., 2009)
BL21(DE3)	Commercially available *E. coli* used for protein expression	Novagen
BL21(DE3) (pET-28a)	BL21(DE3) carrying the pET-28a expression vector, Kan^R^	This study
BL21(DE3) (pHO)	BL21(DE3) carrying the pHO, Kan^R^	This study
MS427	*E. coli* K-12 MG1655 strain mutated in the *agn43* gene	(Kjærgaard et al., 2000a)
MS427(pBAD)	MS427 carrying the pBAD/*Myc*-His A expression vector, Amp^R^	(Wells et al., 2008)
MS427(pIC)	MS427 carrying the pIC, Amp^R^	This study
MS427(pCO4)	MS427 carrying the pCO4, Amp^R^	(Ulett et al., 2007)
Plasmids		
pET-28a	Commercially available expression vector commonly used for protein expression and purification, Kan^R^	Novagen
pBAD/*Myc*-His A	Commercially available expression vector commonly used for protein expression, Amp^R^	Invitrogen
pHO	pET-28a containing the part of the gene responsible for encoding the Eaa passenger domain	This study
pIC	pBAD/*Myc*-His A containing the gene *eaa* from the aEPEC BA92 strain, Amp^R^	This study
pCO4	pBAD/*Myc*-His A containing the gene *agn43A* from the UPEC CFT073 strain, Amp^R^	(Ulett et al., 2007)

### Sequencing, assembly and analysis of the BA92 aEPEC genome

In the present study, the genome of aEPEC BA92 was sequenced using the Oxford Nanopore Technologies (ONT) MinION long-read sequencer. Bacterial genomic DNA was extracted using the QIAamp DNA Micro Kit (Qiagen, NW, Germany) as recommended by the manufacturer. The library was prepared using Rapid sequencing gDNA - barcoding (SQK-RBK004, ONT) and sequenced with a R9.4.1 MinION flow cell using MinKNOW (v22.03) with default settings. Basecalling was performed with Guppy (v5.1, ONT) to obtain long-read FASTQ files.

Further, this data was combined with the Illumina short reads (NCBI accession number: PIJZ00000000), that was obtained in a previous study (Hernandes et al., 2020), for hybrid genome assembly using Unicycler (v0.5.0) (Wick et al., 2017). The genome was annotated using Prokka (v1.14.5) (Seemann, 2014) and Easyfig (v3.0.0) (Sullivan et al., 2011) was employed to illustrate the insertion point of the *eaa*-containing region in the genome of the aEPEC BA92 strain. For comparative purposes, the draft genome sequence of the aEPEC serotype O2:H16 IAL5132 (NCBI accession number: PIKD01000024.1, contig No. 24), that lacks *eaa*, was used (Hernandes et al., 2020). Furthermore, the presence of prophages in the genome of the aEPEC BA92 strain was evaluated using PHASTEST (v3.0) (Wishart et al., 2023).

### *in silico* analysis of the Eaa protein

The amino acid sequence of Eaa was deduced from the nucleotide sequence using CLC Main Workbench 7 software (Qiagen, NW, Germany), and its molecular mass calculated with the Compute pI/MW tool available at the ExPASy website (https://web.expasy.org/compute_pi/) (Gasteiger et al., 2003). The signal peptide cleavage site was predicted using SignalP 6.0 (https://services.healthtech.dtu.dk/services/SignalP-6.0/) (Teufel et al., 2022), and the domains present in Eaa identified using Pfam and InterPro databases (https://www.ebi.ac.uk/interpro/) (Mistry et al., 2021; Paysan-Lafosse et al., 2023).

ClustalW algorithm (Larkin et al., 2007) was used in the MEGA11 software (Tamura et al., 2021) to align Eaa with other characterized autotransporter proteins (**Supplementary Table S1**). The best model to build a maximum likelihood tree was investigated in the IQ-TREE 1.6.12 tool (Nguyen et al., 2015) in combination with the ModelFinder algorithm (Kalyaanamoorthy et al., 2017), which suggested the PMB (Probability Matrix from Blocks model (Veerassamy et al., 2003), with parameters +F+G4 and Gamma shape alpha = 7.1547, as the most appropriate model and parameters to be employed in this analysis. The maximum likelihood was calculated using the UFBoot2 algorithm (Hoang et al., 2018) with 1,000 bootstraps. The resulting Newick file, generated by the IQ-TREE tool, was used as input in the online iTOL tool (https://itol.embl.de/) (Letunic and Bork, 2021) for visualization of the maximum likelihood tree.

The *in silico* Eaa tertiary structure was predicted using ColabFold (https://colab.research.google.com/github/sokrypton/ColabFold/blob/main/AlphaFold2.ipynb) (Mirdita et al., 2022).

### Recombinant plasmids construction

In the present study two recombinant plasmids were constructed: pHO, which corresponds to the pET-28a vector harboring the portion of the *eaa* gene encoding the predicted passenger domain of the autotransporter protein (from nucleotide 62 to 1,415); and pIC, which corresponds to the pBAD/*Myc*-His A vector harboring the entire predicted *eaa* gene (**Table 1**). Both plasmid vectors were extracted from the host bacteria using the QIAprep spin miniprep kit (Qiagen, NW, Germany) and the inserts were prepared by polymerase chain reaction (PCR) amplification performed with the Platinum^®^ Taq DNA Polymerase High Fidelity enzyme (Invitrogen, Vilnius, Lithuania) and using the DNA from the aEPEC BA92 as template. The primers used to amplify the portion of the gene encoding the passenger domain (pET28a-*Bam*HI-F and pET28a-*Xho*I-R) or the entire *eaa* gene (Forward-*Xho*I-F and Reverse-*Kpn*I-R) are described in **Table 2**.

**Table 2:** Primers used in this study.

Primer identification	Primer sequence (5’ → 3’)^a^	PCR conditions		Fragment size (bp)^b^
		Annealing temperature	Extension time	
*eaa*-F	AGGTTTTGTTCGTGAAACTGGA	55°C	45s	534
*eaa*-R	ACAGTTTTTGCTGAGGATGAAAGT			
pET28a-*Bam*HI-F	CGCGCGGGATCCGCATCATTTACTCAAAATATTACAAAAGG	53°C	90s	1,354
pET28a-*Xho*I-R	CGCGCGCTCGAGATGATTCCCTTCAATATCGGTCAAA			
Forward-*Xho*I-F	CGCGCTCGAGATAATAAGGATTCATTATGCTTAATAAAAAAACAATTGC	48°C	150s	2,208
Reverse-*Kpn*I-R	CGCGCGGGTACCTTAAAAGAGGACTCGAACACCAG			
T7 promoter	TAATACGACTCACTATAGGG	49°C	90s	1,393
T7 terminator	GCTAGTTATTGCTCAGCGG			
pBAD-F	ATGCCATAGCATTTTTATCC	44°C	150s	2,451
pBAD-R	GATTTAATCTGTATCAGG			

^a^The enzyme cleavage sites are underlined in the primer sequences; ^b^bp, base pairs.

The part of the gene that encodes the Eaa passenger domain was cloned in *Bam*HI and *Xho*I sites of pET-28a, generating the pHO recombinant plasmid. This allowed the production of the passenger domain carrying a 6xHis-tag on the C-terminal region. The entire *eaa* gene was cloned in *Xho*I and *Kpn*I sites of pBAD/*Myc*-His, generating the pIC recombinant plasmid (**Table 1**). The vectors and the respective inserts were digested with the appropriate enzymes (Invitrogen, Vilnius, Lithuania) and ligated using T4 DNA ligase (Invitrogen, Vilnius, Lithuania) following the manufacturer’s recommendations. The recombinant plasmid pHO was transformed into the BL21(DE3) strain, and the pIC was transformed into the MS427 strain by heat shock (Sambrook et al., 1989).

After the bacterial transformation, the presence of the pHO and pIC recombinant plasmids in the host bacteria cells were verified by PCR performed with the GoTaq Master Mix enzyme (Promega, WI, USA) and using the *eaa*-F and *eaa*-R primers. Subsequently, the DNA sequences cloned in the pHO and pIC recombinant plasmids were confirmed through Sanger sequencing (Sanger et al., 1977) using the primers T7 promoter/T7 terminator and pBAD-F/pBAD-R, respectively. All primers were used at a concentration of 0.34 μM each and are described in **Table 2**.

### Recombinant Eaa-His passenger domain refolding and purification

The production and purification of the Eaa passenger domain was performed as previously described (Chura-Chambi et al., 2022), with some modifications. The BL21(DE3)(pHO) strain was inoculated in 1 L of the rich medium 2-fold HKSII (Jensen and Carlsen, 1990) containing 50 µg/mL of kanamycin and incubated at 37°C. Induction of Eaa passenger domain carrying a His-tag expression was performed by the addition of 0.5 mM of IPTG, when culture reached the optical density of 600 nm (OD600) between 2.0 and 3.0, and cultivation continued at 30°C for 16 h.

The cultures were centrifuged at 4000 x g for 10 min at 4°C, and the resulting pellet was resuspended on lysis buffer (0.1 M Tris HCl- pH 7.0, 5 mM EDTA and 0.1% sodium deoxycholate) and lysed using the Bandelin Sonopuls HD 2070 (Bandelin, BE, Germany). Then, the pellets were washed twice with a buffer containing 0.1 M Tris HCl, 5 mM EDTA and 0.1% of sodium deoxycholate. After the washing step, the pellets were resuspended in a solution containing 50 mM of Tris and 1 mM EDTA (pH 7.0), then centrifuged and suspended in the same buffer. The suspension was transferred to a plastic bag and vacuum sealed (R4-6-40, High Pressure Equipment, USA). After that, it was pressurized at 2,4 kbar for 90 min with a pump (PS-50, High Pressure Equipment, USA) and an air compressor, using an oil-in-water mixture as transmission fluid. After decompression, the samples were centrifuged at 12,000 x g for 15 min, dialyzed in membrane against 25 mM Tris pH 7.0 for 18 h at 4°C under agitation, centrifuged and stocked at -20°C until further use.

The protein was purified using immobilized metal affinity chromatography (IMAC) with a HisTrap FF (GE Healthcare, MA, USA) affinity nickel chromatographic column. This column coupled with the Äkta (GE Healthcare, MA, USA) was balanced using a buffer containing 25 mM Tris pH 7.0 and 150 mM NaCl. Elution was performed using 25 mM Tris pH 7.0, 150 mM NaCl and 1 M imidazol, applying a gradient of 0 to 1 M of imidazol for 30 min (1 mL/min). The eluted fractions were dialyzed against 25 mM sodium phosphate pH 7.0 and later submitted to immunolabeling using anti-His antibodies.

### Anti-Eaa polyclonal serum production

Anti-Eaa polyclonal serum was produced as described by Evans et al. (1975), with some modifications. Of note, pre-immune serum was collected from the rabbit before the immunization protocol. Briefly, a New Zealand White female rabbit was intravenously inoculated with 100 µg of the purified passenger domain of Eaa and 200 µL of Montanide ISA 50 V2 (Butantan Institute, SP, Brazil) as adjuvant. Three immunizations were performed with 15-day intervals. 15 days after the final immunization, approximately 50 mL of blood was collected. The serum fraction was separated and depleted of the complement by heating at 56°C for 30 min. For serum adsorption, 10 glass tubes with 3 mL of LB inoculated with BL21(DE3)(pET-28a) were grown for approximately 18 h, centrifuged and the resulting pellets were stored at -20°C until the time of use. Then, an aliquot of the anti-Eaa serum was mixed with a pellet of the BL21(DE3)(pET-28a) strain and left at gentle agitation for 12 h at room temperature. At the end of this period, the preparations were centrifuged and the anti-Eaa serum was mixed with another frozen pellet of the BL21(DE3)(pET-28a) strain for 12 h. These cycles were repeated until the 10 frozen pellets were used. Finally, the anti-Eaa serum was stored at -20°C. The anti- Eaa serum production protocol was approved by the Ethics Committee on Animal Use of the Butantan Institute (CEUAIB protocol: 9452010316).

### Immunoblotting with anti-Eaa serum

The purified recombinant protein was submitted to SDS-PAGE 12% (Laemmli, 1970) under denaturing conditions, transferred to a nitrocellulose membrane (Bio-Rad, CA, USA) and examined with the anti-Eaa serum. Then, the membrane was incubated in 10% skim milk (diluted in PBS: phosphate buffered saline pH 7.4) at 4°C for 16 h, washed three times with PBS supplemented with 0.05% Tween-20 (PBS-T), and, further, incubated with anti-Eaa serum (1:200) at room temperature for 1 h. Then, the membrane was washed with PBS-T and incubated with goat anti-rabbit IgG secondary antibodies conjugated with peroxidase (Sigma-Aldrich, HE, Germany) (1:10,000) for 1 h at room temperature. After washing with PBS-T, the membrane was developed using SuperSignal West Pico PLUS Chemiluminescent Substrate (Thermo Fisher Scientific, MA, EUA) and visualized using the transilluminator Amersham Imager 600 (GE Healthcare, MA, USA).

### Immunogold labeling

Bacterial strains aEPEC BA92, MS427(pBAD) and MS427(pIC) were cultivated overnight at 37°C in LB broth supplemented with 0.2% L-arabinose (Sigma-Aldrich, HE, Germany) and 100 μg/mL ampicillin, when appropriate. Bacterial cultures were then centrifuged (2,348 x g for 5 min) and the supernatant was discarded. After four washings (2,348 x g for 5 min centrifugation) with PBS, preparations were fixed with 4% paraformaldehyde in PBS (Sigma-Aldrich, HE, Germany) for 30 min at room temperature. Preparations were then washed four times with PBS containing 0.2% bovine serum albumin (BSA) (Thermo Fisher Scientific, MA, USA) (PBS-BSA), and blocked in the same solution for 60 min at room temperature. After centrifugation to remove PBS- BSA, preparations were incubated with rabbit pre-immune serum or anti-Eaa serum, diluted (1:25) in PBS, for 18 h at 4°C. After this period, the preparations were washed five times with PBS-BSA and incubated with a goat anti-rabbit serum conjugated with 10 nm colloidal gold particles (Sigma-Aldrich, HE, Germany) diluted (1:25) in PBS at room temperature for 5 h. After five washings with PBS, the preparations were applied onto 200 mesh copper grids (Electron Microscopy Sciences, PA, USA) previously prepared with 0.5% Formvar (Sigma-Aldrich, HE, Germany) in chloroform (Merck, HE, Germany), and left for 2 min. After removing the excess of liquid and drying at room temperature, the preparations were analyzed using a LEO 906E transmission electron microscope (Zeiss, BW, Germany) operating at 80 kV in the Structural and Functional Biology Laboratory of the Butantan Institute.

### Autoaggregation assay

Autoaggregation assays were carried out as previously described (Kjærgaard et al., 2000b), with modifications. Briefly, the bacterial strains were incubated at 37°C with agitation for 18 h in 40 mL of Brain Heart Infusion (BHI) broth (Oxoid, Basingstoke, England) supplemented with 0.2% L-arabinose and 100 µg/mL ampicillin. The cultures were then centrifuged at 5,000 x g for 10 min and the supernatant discarded. The resulting pellet was resuspended in sterile BHI, containing 0.2% L-arabinose and 100 µg/mL ampicillin, and the OD600 adjusted to 1.0. Then, 10 mL of each preparation were divided into two glass tubes with 5 mL each, one of which was kept static during all the assay, while the other was homogenized before the OD600 readings. The autoaggregation assay was carried out for a total of 2 h. After 20 min, 200 µL of each preparation was collected from the very top and transferred to a 96-well plate. The OD600 was measured using the BioTek Epoch 2 with the Gen5 software (Agilent, CA, USA).

At the end of the autoaggregation assay, 10 μL of the bottom of each culture was collected and transferred to a glass microscope slide, which was heat-fixed and subsequently stained with 4’,6-Diamidino-2-phenylindole (DAPI; Thermo Fisher Scientific, MA, USA) (diluted 1:500 in PBS) for 20 min and kept protected from light during this period. Then, the slides were washed by immersion in distilled water, dried, and analyzed under a BX60 fluorescence microscope (Olympus, Kanto, Japan).

### Biofilm production on abiotic surfaces

The ability of the bacterial strains to produce biofilm on polystyrene plates was performed as previously described (Oliveira-Garcia et al., 2003), with modifications. In each well of a 96-well microplate (Tissue Culture Plates 96 well – K12-096, Kasvi, PR, Brazil), 200 μl of LB were added containing 2% D-mannopyranoside (Sigma-Aldrich, HE, Germany), 0.2% L-arabinose and 100 μg/mL ampicillin, as well as 10 μL of overnight bacterial cultures. After 24, 48 or 72 h of incubation at 37 °C, each well was washed with 200 μL of PBS and then fixed with 200 μL of 3% formaldehyde for 1 h. After, the wells were washed with distilled water and the preparations stained with 1% crystal violet for 20 min. After the staining step, the wells were washed with distilled water and the plate was dried at room temperature for approximately 2 h. The crystal violet was solubilized with 200 μL of methanol for 10 min and the OD570 measured in an ELISA BioTek Epoch 2 reader with the Gen5 software.

Additionally, we also investigated the ability of the bacterial strains to produce biofilm on glass surfaces. Sterile glass coverslips were introduced into 24-well polystyrene plates (Tissue Culture Testplate 24 – 92024, TPP, SH, Switzerland). Subsequently, 950 µL of LB, containing 2% D-mannopyranoside, 0.2% L-arabinose and 100 µg/mL ampicillin, were added as well as 50 µL of the bacterial cultures. The plates were then incubated at 37°C for 24, 48 or 72 h. After the distinct periods of incubation, the wells were washed with 1 mL of PBS, followed by fixation with 1 mL of 3% formaldehyde for 1 h. Then, the wells were washed with distilled water and the coverslips were transferred to a novel 24-well plate. The glass coverslips were stained with 1 mL of 0.1% crystal violet for 20 min, washed with distilled water and dried at room temperature. The crystal violet was then resuspended with 33% glacial acetic acid for 10 min and the OD570 measured in a BioTek Epoch 2 ELISA reader with the aid of the Gen5 software.

Biofilm production on polystyrene was performed in three biological replicates, carried out on different days, with five technical replicates being performed in each assay. For the glass surfaces tests, three technical replicates were performed instead.

### Eaa binding assay to extracellular matrix components

Eaa binding to ECM components was carried out as previously described (Salazar et al., 2014), with modifications. Collagens type I, III, (Corning, NI, USA), IV and V, cellular and plasma fibronectin, laminin, fibrinogen, vitronectin, and the negative controls BSA and fetuin (Sigma-Aldrich, HE, Germany), diluted at 10 μg/mL in PBS, were immobilized in 96-well plates (96 Well EIA/RIA Assay Microplate – high binding surface – 3590, Corning, NI, USA) and incubated at 4°C for 18 h. The wells were washed three times with phosphate buffered saline – 0.05% Tween 20 (PBS-T) and non-specific binding sites were blocked using 100 µL of 1% BSA for 2 h at 37°C. Then, 100 µL of recombinant Eaa (0.1 μM) diluted in PBS were added, and incubation proceeded for 1.5 h at 37°C. After three washes with PBS-T, anti-Eaa serum produced in rabbit (diluted 1:1,000 in PBS) was added and incubated for 1 h at 37°C. Subsequently, peroxidase conjugated goat anti-rabbit IgG (diluted 1:5,000 in PBS) (Sigma-Aldrich, HE, Germany), was added and incubated for an additional hour at 37°C. The wells were washed three times and *o*-phenylenediamine (0.04%) in citrate phosphate buffer (pH 5.0) plus 0.01% H_2_O_2_ was added. The reaction was carried out for 15 min and stopped with 50 µL of H_2_SO_4_ 8N. The absorbance was read at 492 nm using the Multiskan EX device (Thermo Fisher Scientific, MA, USA).

To further explore the interaction of Eaa with fibronectin, a dose-dependent binding using up to 2 μM of recombinant protein was carried out. Furthermore, mapping of the fibronectin interacting domain with the Eaa passenger domain was also evaluated. In this case, F30 (heparin binding domain – HBD) (Sigma-Aldrich, MO, USA) and F45 (gelatin binding domain – GBD) (Sigma-Aldrich, MO, USA) fibronectin domains were used, and the binding assay was performed essentially as described above. All assays were carried out with two technical and three biological replicates.

### Statistical analyses

To identify statistical differences among the bacterial strains used in the autoaggregation assay, second-degree polynomial regressions adjustments were performed for all replications and subsequent analysis of variance were performed for each parameter. Biofilm data were analyzed through ANOVA followed by Tukey and ECMs binding was analyzed by ANOVA followed by Dunnett’s test. The dose-dependent binding assays were analyzed through multiple *t* tests. *P* < 0.05 was adopted to consider the differences observed between the groups as significant.

## Results

### Identification of a novel autotransporter adhesin-encoding gene in aEPEC strains of serotype O2:H16

Initially, we used a dataset generated in a previous study (Hernandes et al., 2020) to investigate the possible occurrence of a set of genes unique to aEPEC of serotype O2:H16 when compared to a global collection of EPEC isolates. This analysis demonstrated that 31 genes were present only in aEPEC strains of this serotype (**Supplementary Table S2**). Among them we highlighted one predicted to encode an uncharacterized autotransporter protein. This gene was found in 6 (85.7%) of the 7 aEPEC strains of serotype O2:H16 analyzed (**Supplementary Table S2**) with 100% coverage and identity. Importantly, based on its predicted adhesive properties, this novel autotransporter protein was named in the present study as EPEC Autotransporter Adhesin (Eaa).

**Figure 1:**
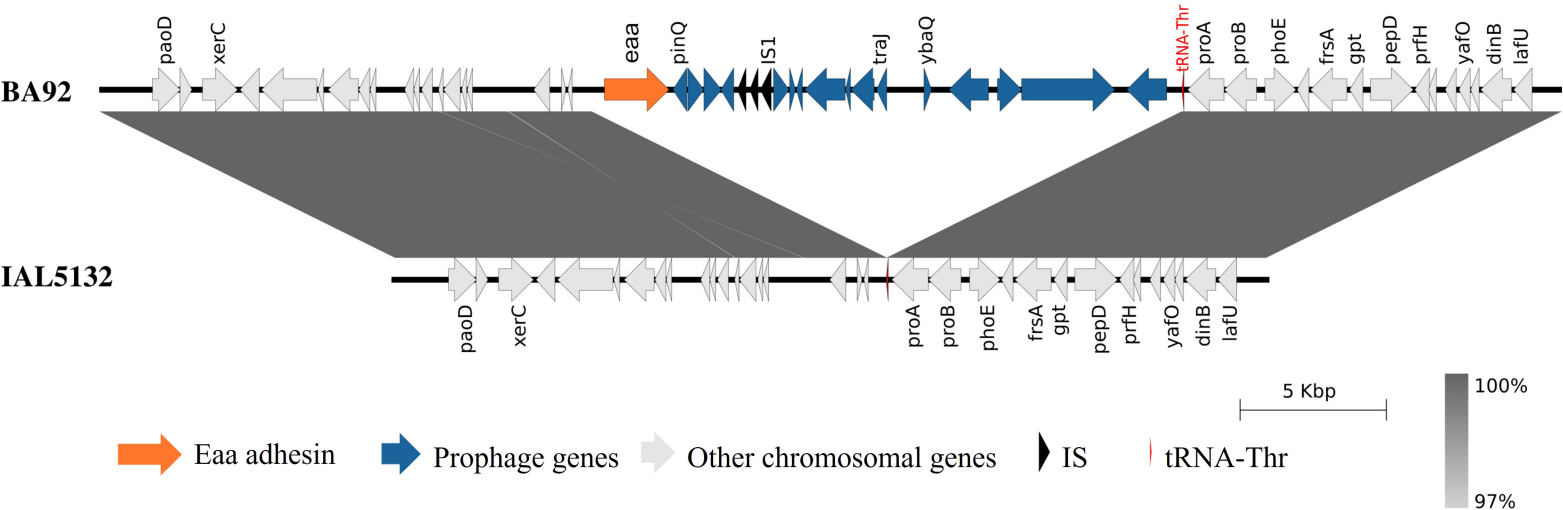
Visualization of the chromosomal region that harbors the *eaa* gene in the aEPEC BA92 strain. A comparative genomic analysis of the chromosome of the aEPEC BA92 (NCBI accession number: CP176406) with the draft genome sequence of the aEPEC IAL5132 (NCBI accession number: PIKD01000024.1, contig No. 24) revealed that the *eaa* gene is located on a chromosomal region with 17,014 base pairs organized in 20 coding sequences. This chromosomal region is inserted downstream to the gene encoding the transfer RNA for the amino acid threonine (tRNA-Thr). The protein ID for the Eaa is XWX38401.1.

Subsequently, to better understand the genetic context in which the gene encoding the autotransporter protein Eaa is inserted in the genome of aEPEC of serotype O2:H16, the strain BA92 was selected. The ONT MinION long read sequence generated a total of 450,101 reads with a coverage of 429.56 times. The hybrid genome assembly (using the MinION long reads combined with the Illumina short reads) showed that the predicted genome size of the aEPEC BA92 was 5,134,725 bp, with 50.22% of GC content and composed of the chromosome and 5 distinct plasmids (**Supplementary Table S3**).The *eaa* gene is located in the chromosome of this strain in a genomic region identified as a prophage (**Figure 1**; **Supplementary Table S4**). Using the strain IAL5132 (an aEPEC strain of serotype O2:H16 that lacks the *eaa* gene) as reference, we could determine that the *eaa*-chromosomal containing region present in the aEPEC BA92 strain is 17,014 base pairs in length, organized in 20 coding sequences, including *eaa*, and inserted upstream to the DNA sequence encoding for the transfer RNA of the amino acid threonine (**Figure 1**). All genes present in the chromosomal region containing the prophage harboring the *eaa* gene are listed in **Supplementary Table S5**.

### The autotransporter protein Eaa is a novel member of the AIDA-I family

**Figure 2:**
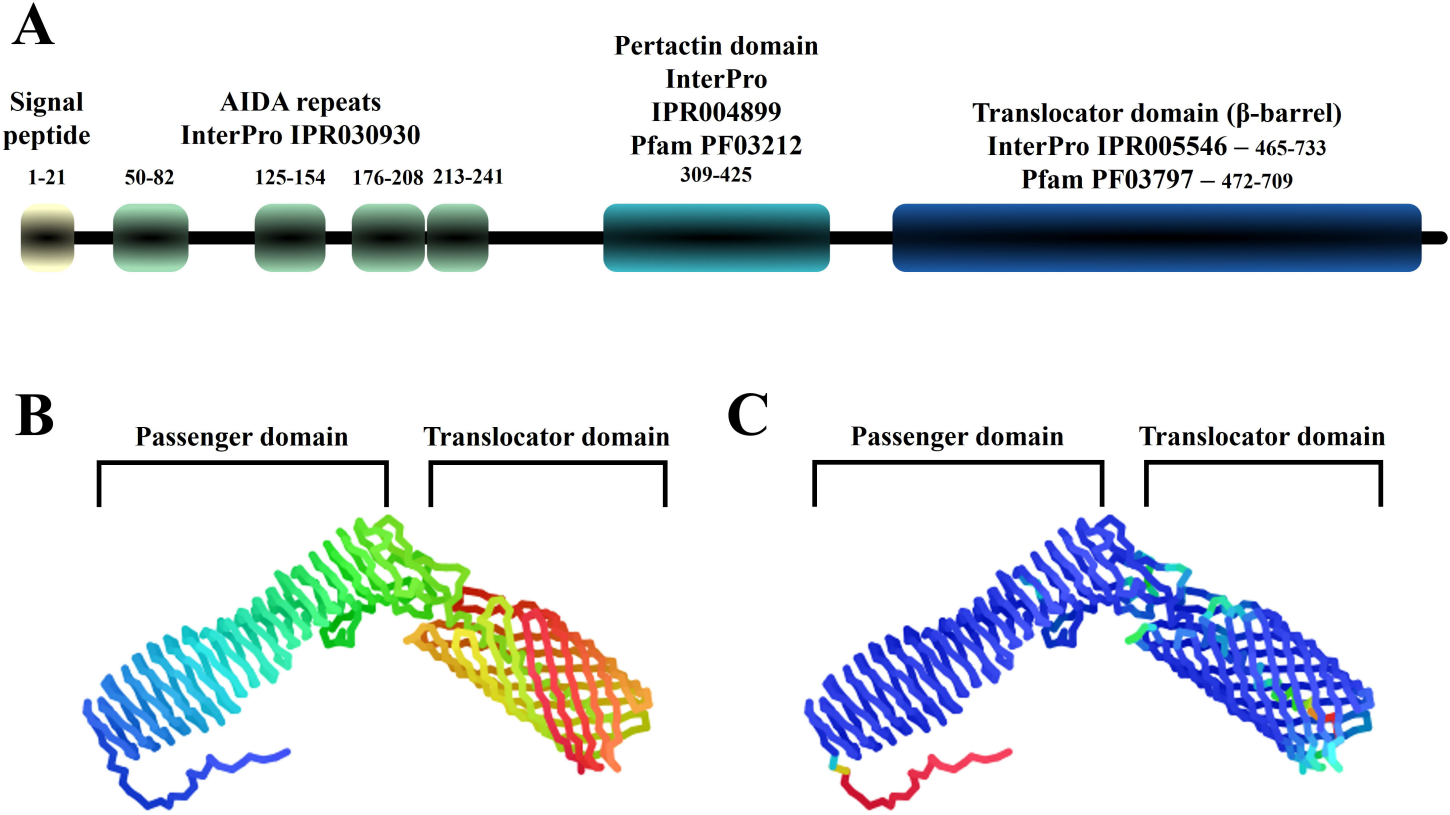
Protein domains and tertiary structure of the Eaa protein. This image illustrates known protein domains found in Eaa according to InterPro and Pfam databases. **(A)** and structural prediction according to ColabFold **(B, C)**. **(A)** the signal peptide, four AIDA domain repeats, a pertactin domain and the translocator domain (β- barrel) are highlighted. **(B)**, the N- and C-terminal regions are respectively shown in blue and red, while in **(C)** the protein is colored according to its lDDT (local Distance Difference Test) score which is categorized as follows: Dark blue >90, light blue = 80, green = 70, yellow = 60 and red <50.

The deduced amino acid sequence of the complete Eaa products has 733 amino acids in length with a predicted molecular mass of 79 kDa. The signal peptide of the Eaa protein was identified as the first 21 amino acids (**Figure 2A**). Moreover, four AIDA- repeats, one pertactin domain and one β-barrel translocator domain were also identified (**Figure 2A**). As expected, Eaa is predicted to be a monomeric protein with its passenger and the β-barrel translocator domains located in the N-terminal and C-terminal regions, respectively (**Figures 2B, C**).

**Figure 3:**
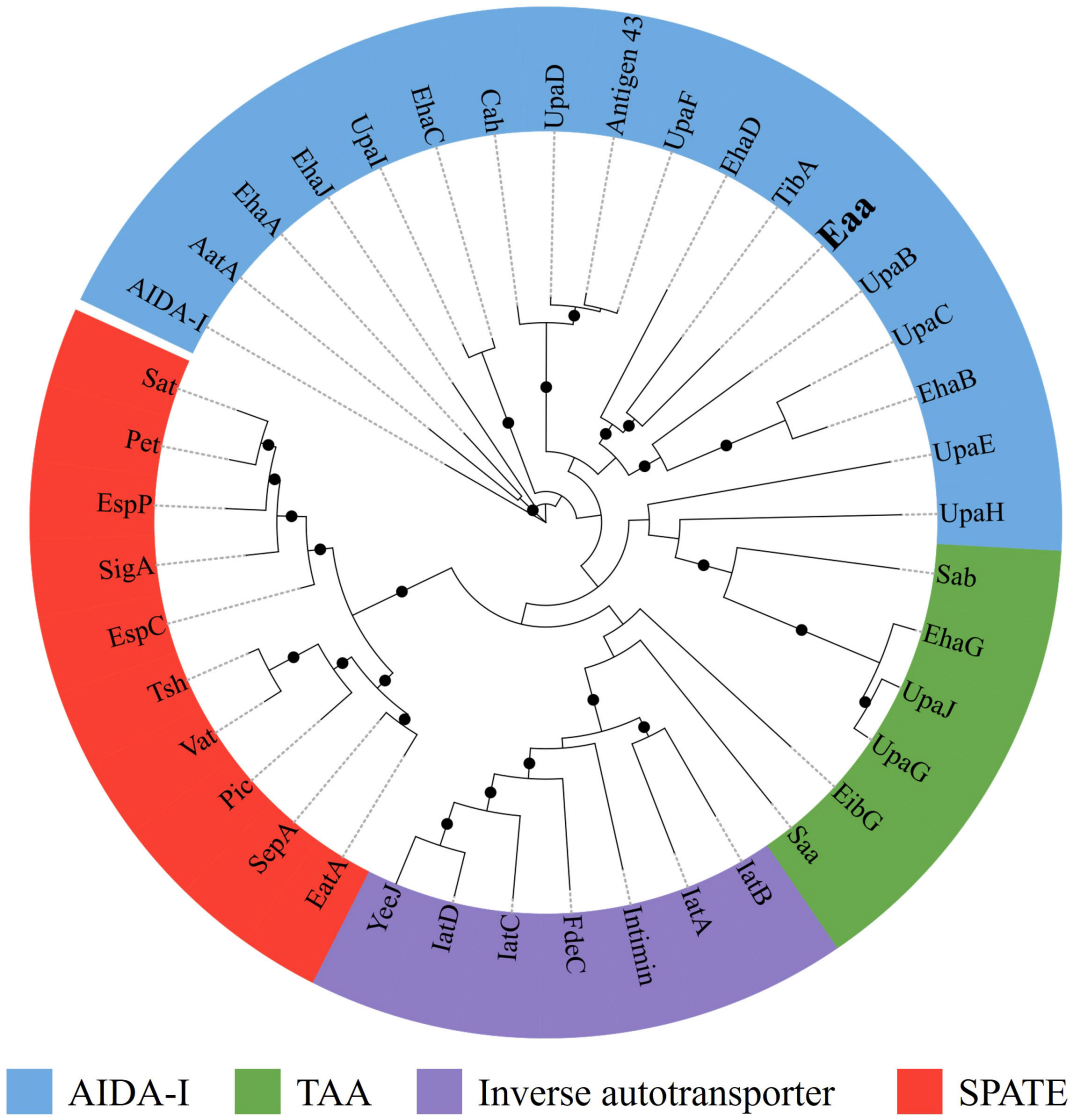
The novel autotransporter protein Eaa belongs to the AIDA-I family of adhesins. The amino acid sequences of several well-characterized autotransporter proteins (**Supplementary Table S1**), were aligned using ClustalW in MEGA 11 and the maximum likelihood tree was calculated using IQ-Tree 1.6.12 along with the UFBoot2 algorithm using 1,000 bootstraps and the PMB model. The resulting Newick file was visualized using the iTOL software. The maximum likelihood tree demonstrated that Eaa belongs to the AIDA-I family and is closely related to the adhesins TibA and EhaD, first described in the enterotoxigenic (ETEC) and enterohemorrhagic (EHEC) *E. coli* pathotypes, respectively. The nodes with ultrafast bootstrap support values >80% are labeled with a black circle. The novel autotransporter protein Eaa is highlighted in bold.

Furthermore, a maximum likelihood analysis, comparing the amino acid sequence of some representative autotransporter proteins (**Supplementary Table S1**) from the four autotransporter families, indicated that Eaa belongs to the AIDA-I family and is closely related to the adhesins TibA and EhaD (**Figure 3**). Furthermore, an amino acid alignment between Eaa and TibA demonstrated that these two proteins present 41.1% similarity and 26.2% identity (**Supplementary Figure S1**).

### Eaa is an outer membrane protein

First, the production of the autotransporter protein Eaa was investigated by immunoblotting using the anti-Eaa serum. The SDS-PAGE results showed the production of a 79 kDa protein in the cultures of aEPEC BA92 and MS427(pIC) strains (**Supplementary Figure S2**), thus indicating the Eaa production by these bacteria.

**Figure 4:**
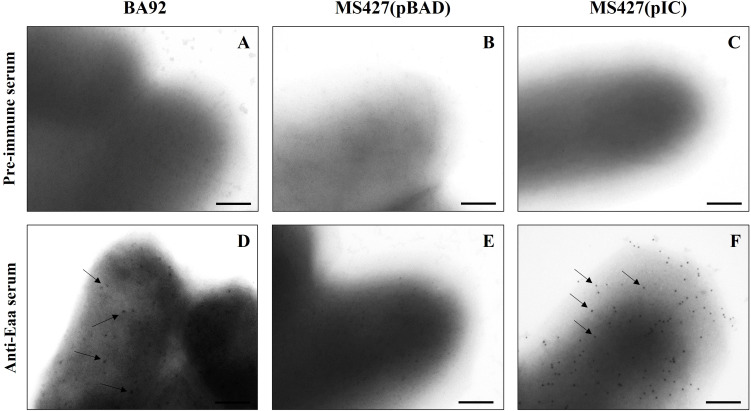
Identification of Eaa on the bacterial cell surface of aEPEC BA92 and MS427(pIC) strains. Immunogold-labelling (IGL) preparations using pre-immune **(A-C)** or anti-Eaa **(D-F)** sera, produced in rabbit (primary antibodies), and goat anti-rabbit serum conjugated with 10 nm colloidal gold particles (secondary antibody) were analyzed by transmission electron microscopy. IGL using anti-Eaa serum **(D-F)**, demonstrated that Eaa is present on the bacterial cell surface of both BA92 (wild- type) and MS427(pIC) strains, as indicated by arrows **(D, F)**. Note that the preparations were not negatively stained, and that gold particles were not observed on the surface of the MS427(pBAD) strain **(E)**, used as a negative control in this assay, nor in any of the preparations incubated with the pre-immune serum **(A-C)**. Bars, 200 nm.

Subsequently, an immunogold-labelling transmission electron microscopy was performed to verify whether the autotransporter Eaa protein would be able to reach the extracellular environment and remain anchored in the outer membrane. This analysis demonstrated that Eaa was detected on the surface of the aEPEC BA92 and MS427(pIC) strains, but not on the surface of the MS427(pBAD) strain, used as a negative control in this assay (**Figure 4**).

### Eaa mediates bacterial autoaggregation and biofilm formation

To investigate the participation of Eaa in the autoaggregation and biofilm formation phenotypes, the MS427 strain (that correspond to the K-12 *E. coli* MG1655 deleted in the *agn43* gene), was used as a host bacteria for three different plasmids, as follows: pBAD/*Myc*-His A, pIC (that corresponds to the pBAD/*Myc*-His A vector harboring the *eaa* gene) and pCO4 (that corresponds to the pBAD/*Myc*-His A vector harboring the *agn43* gene), as described in **Table 1**. Of note, the *agn43* gene encodes an autotransporter adhesin, termed Antigen 43, whose role in bacterial-bacterial autoaggregation and biofilm formation was already documented in the literature (Kjærgaard et al., 2000a, 2000b). Considering that, MS427(pBAD) and MS427(pCO4) were employed as negative and positive controls, respectively.

**Figure 5:**
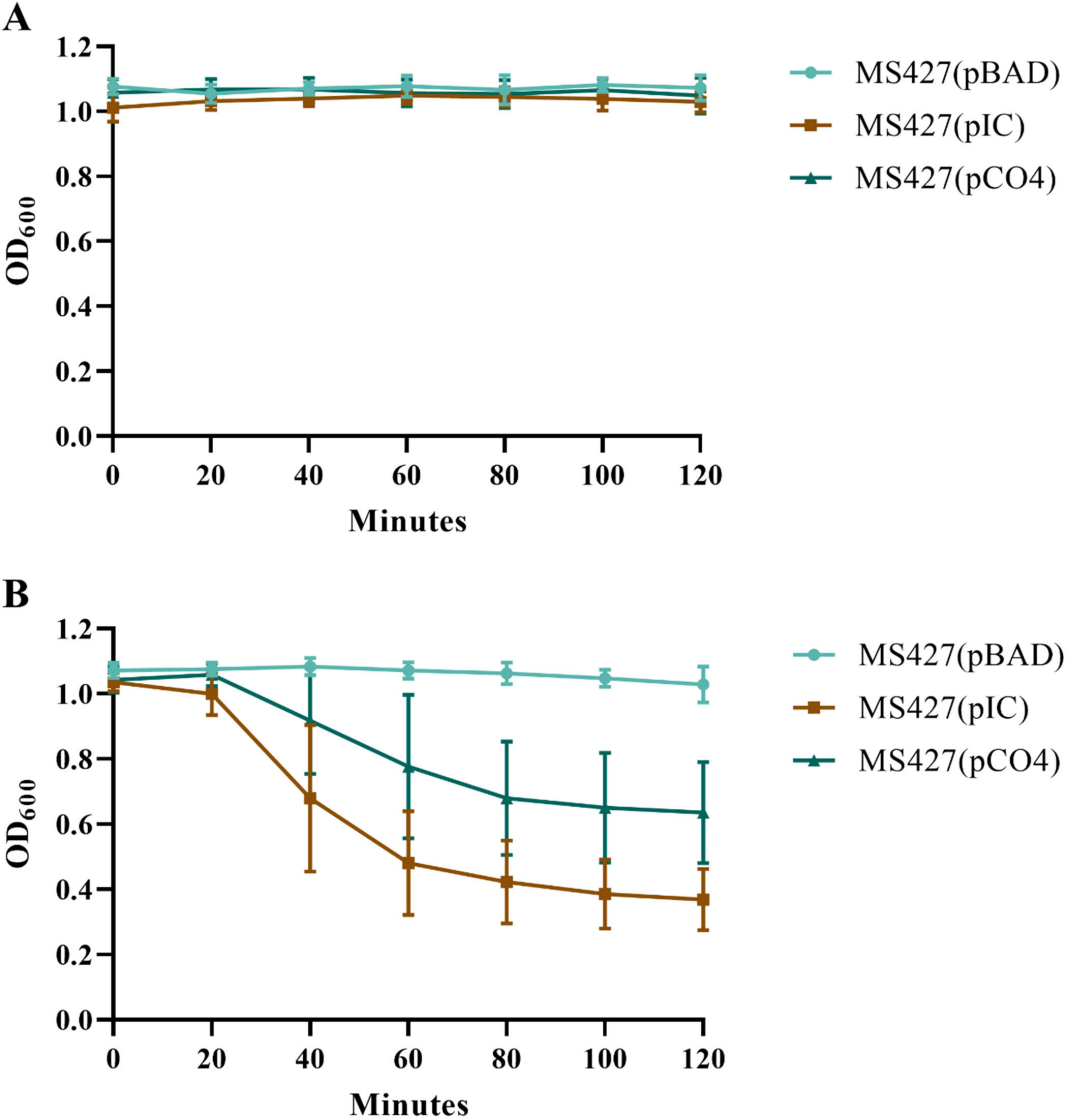
Eaa promotes bacterial autoaggregation. The autoaggregation assay was carried out under two experimental conditions: **(A)** with homogenization of the bacterial cultures and **(B)** without homogenization of the bacterial cultures before reading the optical density at 600 nm (OD600). Note, in panel **(B)**, that the OD600 of the bacteria that produce Eaa, MS427(pIC), as well as the Antigen 43, MS427(pCO4), progressively decrease throughout the autoaggregation assay. Statistical difference between MS427(pBAD), used as a negative control in this assay, and MS427(pIC) was observed at all time points (*P*<0.001). Error bars show standard deviation.

In the autoaggregation assay, we observed a progressive decrease of the OD600 at each reading for two of the strains tested: MS427(pIC) and MS427(pCO4); while for the MS427(pBAD) strain, used as a negative control, the OD600 remained constant throughout the assay (**Figure 5**). In all periods in which the DO600 was measured, the strains MS427(pIC) and MS427(pCO4) differed statistically from the negative control, as well as from each other (*P*<0.001).

**Figure 6:**
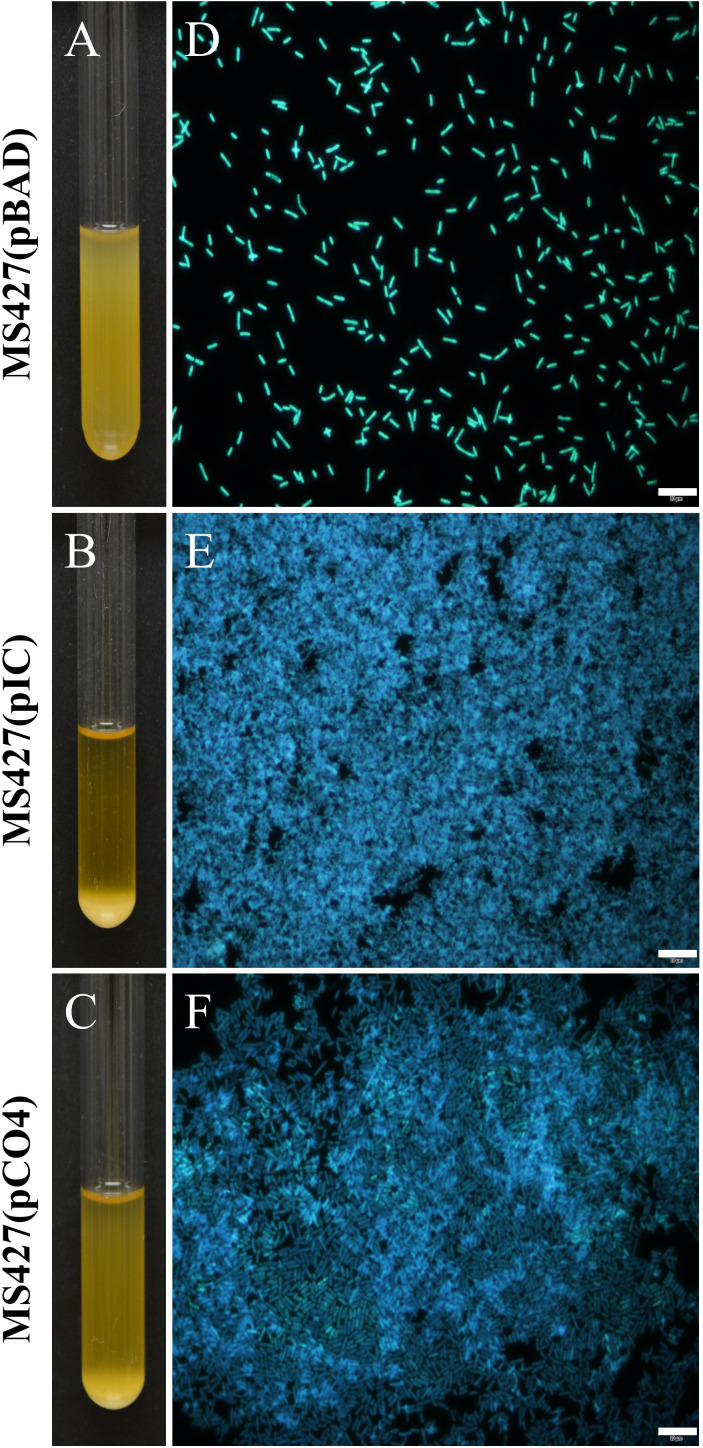
Eaa promotes the formation of large bacterial aggregates. **(A-C)** show the settling patterns of the cultures left statically for 2 hours at room temperature, while the **(D-F)** are fluorescence microscope images of bacterial sediments stained with DAPI. Note in panels **(B, C)** the large amount of bacterial cells sedimented at the bottom of the test tube from the broth cultures of the strains MS472(pIC) and MS427(pCO4), that produce the autotransporter proteins Eaa and Antigen 43, respectively. The bacterial aggregates induced by the production of Eaa and Antigen 43 can be visualized in **(E, F)**, respectively, while in **(D)** only the occurrence of isolated bacteria is observed. Bars = 10 µm.

To confirm the hypothesis that OD600 decreased due to bacterial autoaggregation, an aliquot of the bacterial pellet, formed at the bottom of the test tube at the end of the assay, was collected and subjected to immunofluorescence microscopy. While for the MS427(pBAD) strain only isolated bacteria were observed, the MS427(pIC) and MS427(pCO4) strains formed large bacterial aggregates (**Figure 6**). Taken together, these data demonstrated the ability of Eaa in mediating bacterial autoaggregation.

**Figure 7:**
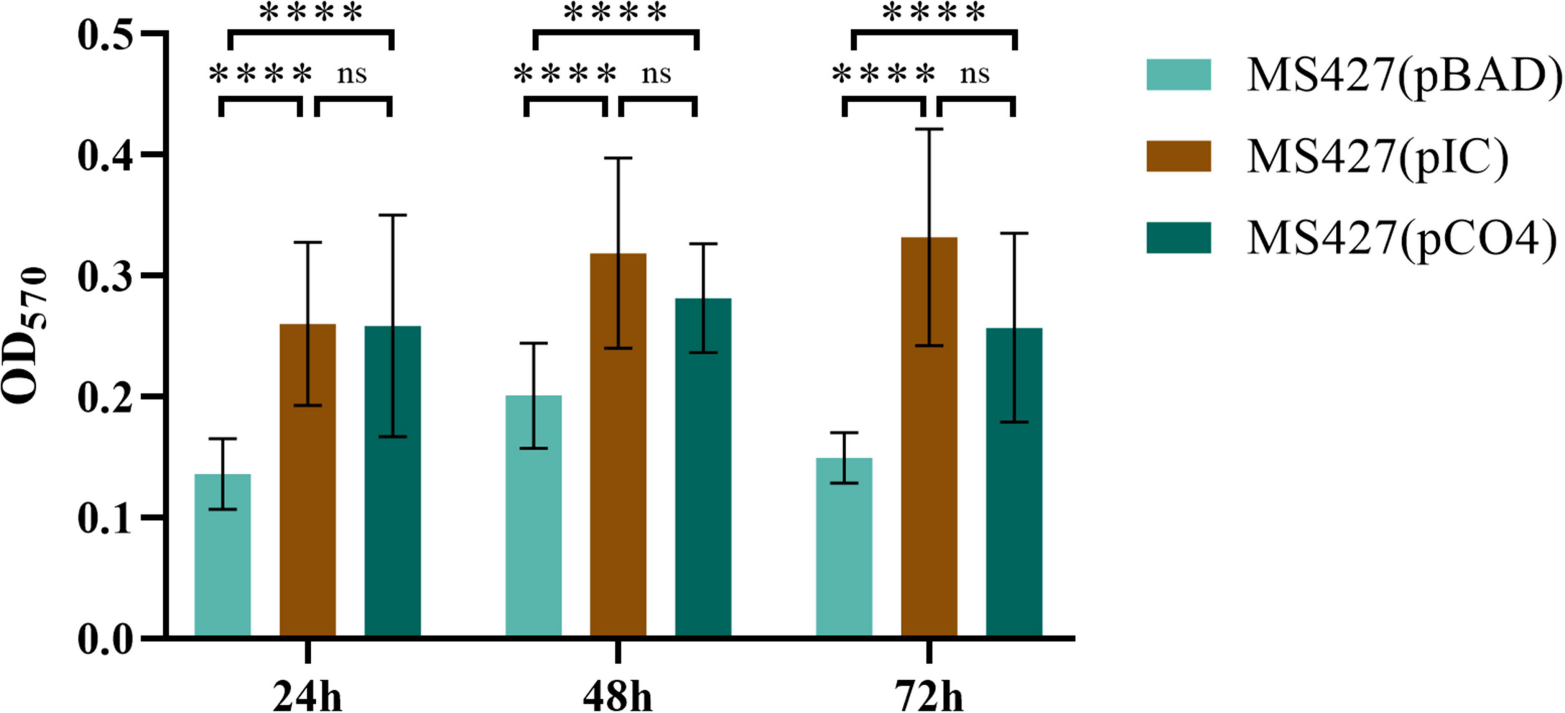
Eaa increases biofilm formation on polystyrene. Biofilm production was evaluated on polystyrene surface in three periods of time: 24, 48 and 72 hours. Note that the bacteria producing the autotransporter proteins Eaa, MS427(pIC), as well as the Antigen 43, MS427(pCO4), produce significantly more biofilm than the host bacteria carrying the pBAD/*Myc*-His A expression vector. Error bars show standard deviation. ****P<0.0001 and ns, non-significant.

Biofilm formation assays employing the Eaa-producing strain (MS427(pIC)), in comparison to the negative (MS427(pBAD)) and positive (MS427(pCO4)) controls, were preformed to investigate the involvement of Eaa in biofilm formation. The MS427(pIC) produced significantly more biofilm on polystyrene than the negative control MS427(pBAD) in all periods of times tested (24, 48 and 72 h), similarly to the observed with the positive control MS427(pCO4) (*P*<0.0001). On the other hand, no statistical difference (*P*>0.05) was observed between the MS427(pIC) and MS427(pCO4) strains that produce the autotransporter proteins Eaa and Antigen 43, respectively (**Figure 7**). No increase in biofilm production was evidenced in the MS427(pIC) and MS427(pCO4) when compared to the negative control MS427(pBAD) in assays performed on glass coverslips surface (data not shown).

### Eaa binds to extracellular matrix and plasma components

**Figure 8:**
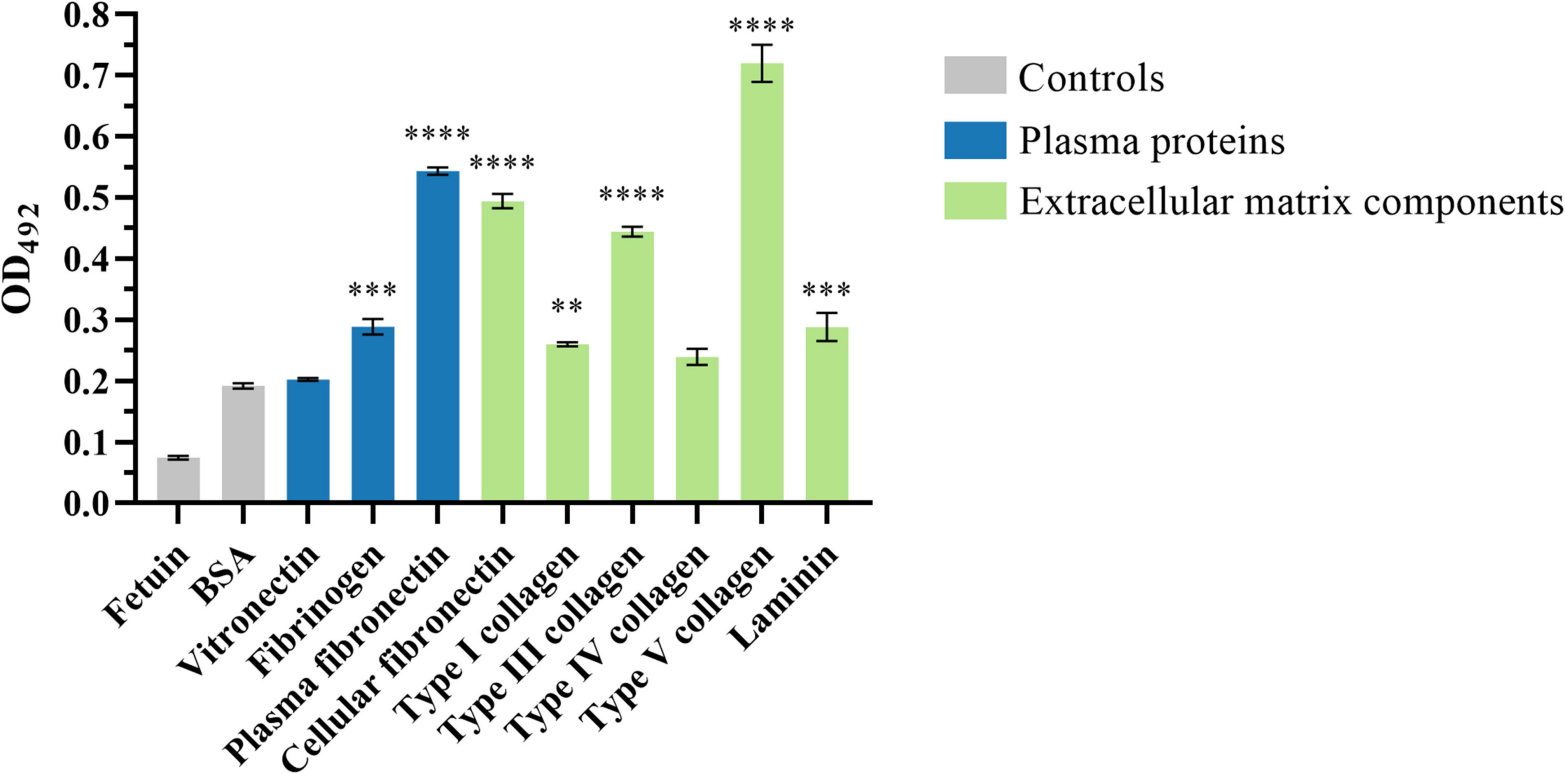
Recombinant Eaa binds to several plasma and extracellular matrix (ECM) components. For this assay, wells were coated with 1 μg of the ECM components followed by the addition of 0.1 μM of recombinant Eaa per well. The binding assay to ECM components demonstrated that the recombinant Eaa-His protein binds to fibrinogen, plasma and cellular fibronectin, type I, III and V collagen and laminin. Bovine serum albumin (BSA, nonglycosylated attachment-negative control protein) and fetuin (highly glycosylated attachment-negative control protein) were used as negative controls in this binding assay. Optical densities were taken at 492 nm. Data represent the mean ± standard error of three independent experiments. Statistical differences relative to the values obtained for BSA are shown. **P=0.0097, ***P=0.0003 and ****P<0.0001.

**Figure 9:**
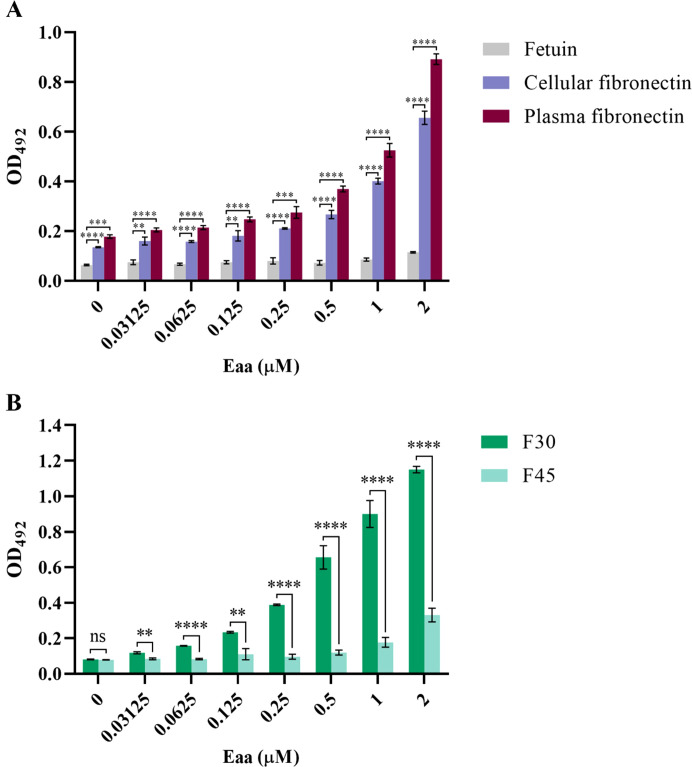
Eaa dose-dependently binds to cellular and plasma fibronectin through its heparin-binding domain (F30). Binding of recombinant Eaa (0 to 2 µM) to cellular and plasma fibronectin **(A)**, as well as to F30 (Heparin Binding Domain, HBD) and F45 (Gelatin Binding Domain, GBD) fibronectin domains **(B)** was assessed by an ELISA- based assay. Wells were coated with 1 µg of intact fibronectin **(A)** or with F30 and F45 fragments **(B)** and increasing amounts of recombinant Eaa (0 – 2 µM) were added to each well. Optical densities were taken at 492 nm. Note that the recombinant Eaa-His protein binds to cellular as well as plasma fibronectin in a dose-dependent manner **(A)**; and binds more efficiently to the F30 fibronectin domain **(B)**. Data represents the mean ± standard error of three independent experiments. Statistical differences relative to the values obtained for fetuin are shown in **(A)**. Statistical comparisons were performed using multiple *t* tests. **P ≤ 0.01; ***P ≤ 0.001; ****P ≤ 0.0001 and ns, non-significant.

The ability of the passenger domain of the autotransporter protein Eaa to bind to several ECM and plasma components was investigated. Purified recombinant Eaa (aminoacids 22 - 470) bound to most of the macromolecules tested, i.e. fibrinogen, plasma and cellular fibronectin, laminin and type I, III and V collagens, with especially pronounced binding to collagen V and fibronectin (**Figure 8**). Adhesion to plasma and cellular fibronectin was further explored on a quantitative basis. A dose-dependent binding to both ECM molecules was observed at increasing concentrations of Eaa (**Figure 9A**). Furthermore, the fibronectin domains responsible for binding were mapped using purified F30 (heparin binding domain, HBD) and F45 (gelatin binding domain, GBD). Eaa dose-dependently interacted with the HBD of fibronectin as shown in **Figure 9B**.

## Discussion

Since aEPEC does not produce BFP, several studies in the literature have focused on the search for additional adhesins that, in association with intimin, could allow this pathogen to adhere to host cells, as well as abiotic surfaces. To the best of our knowledge, the only adhesin that has been first identified in aEPEC so far was the locus for diffuse adherence (Lda) (Scaletsky et al., 2005). The Lda afimbrial adhesin was identified in an aEPEC strain of serotype O26:H11 and mediates diffuse adherence on HEp-2 cells when produced by a K-12 *E. coli* strain (Scaletsky et al., 2005). Furthermore, many other studies have reported the occurrence of several adhesin-encoding genes, previously identified in other DEC pathotypes, in aEPEC strains (Scaletsky et al., 2009; Gomes et al., 2011; Hernandes et al., 2011; Vieira et al., 2016; Munhoz et al., 2018). However, whether aEPEC can produce these adhesins and if they have any role in adhesion during *in vivo* infection are questions that have not yet been answered. Contributing to this scenario, in the present study we identified the novel autotransporter adhesin, Eaa. We have demonstrated a role for Eaa in bacterial autoaggregation, biofilm formation and binding to several ECM components.

Autoaggregation is a phenotype that occurs when self-recognizing structures, present on the outer membrane of the bacteria cells, interact with each other leading to the formation of large bacterial aggregates. It has been reported in the literature that the autoaggregation phenotype favors biofilm production (Trunk et al., 2018). Furthermore, the formation of these bacterial aggregates may facilitate evasion of the immune system by hindering phagocytosis of these bacterial aggregates by granulocytes and/or promoting resistance to hydrogen peroxide (H_2_O_2_) within these phagocytic cells (Fexby et al., 2007). Similar to Eaa, several diverse autotransporter proteins have been described mediating the autoaggregation phenotype in distinct pathogenic or commensal *E. coli* strains, such as AIDA-I, TibA, Antigen 43 and Cah (Sherlock et al., 2004, 2005; Kjærgaard et al., 2000b; Torres et al., 2002). Considering the bacterial autoaggregation phenotype, two interesting situations have already been described, such as the formation of *E. coli* bacterial clusters due to the AIDA-I and Antigen 43 interaction (Sherlock et al., 2004), as well as the formation of interspecific bacterial aggregates (*E. coli* and *Pseudomonas fluorescens*) mediated by Antigen 43 (Kjærgaard et al., 2000a). However, whether Eaa can interact with other autotransporter adhesin from the AIDA-I family or mediate interspecific bacterial aggregation are questions that deserve to be answered in the future.

Another major adherence mechanism that can be mediated by autotransporter adhesins is the formation of biofilms, which are complex masses comprised mostly (about 90%) of extracellular polymeric substances (EPS) and microorganisms (Wells et al., 2008; Allsopp et al., 2012; Battaglioli et al., 2018). The biofilm phenotype can lead to increased bacterial adherence to host cells or abiotic surfaces and serves as a mechanism of resistance to environmental stress, such as temperature and pH, as well as chemical factors, such as antimicrobial compounds and bacteriocins. Due to these properties, biofilms can be associated with persistent infections (Flemming et al., 2016). In the present study, we demonstrate that Eaa mediates biofilm formation on polystyrene, but not on glass. These data are consistent with previous studies that evaluated biofilm formation by *E. coli* isolates on distinct abiotic surfaces, showing that biofilm development can vary significantly depending on the material used in these assays (Gomes et al., 2015; Bang et al., 2014). In fact, some reports associated aEPEC with cases of persistent diarrhea, which could be linked to the capacity of an aEPEC isolate to form a biofilm in the intestinal tract, thus prolonging the colonization and disease (Ochoa et al., 2008; Ochoa and Contreras, 2011; Nguyen et al., 2006; Moore et al., 2010; Afset et al., 2004).

The ECM of the gastrointestinal tract is composed of a complex of proteins and polysaccharide molecules which are divided into two distinct parts that are intimately interconnected: the basement membrane (BM) and the interstitial matrix (IM). The BM is a layer of approximately 100 nm interleaved between the epithelium and mesenchyme of lamina propria and consists predominantly of collagen type IV, laminins, nidogens, and perlecan. The IM is located beneath the BM and acts as an important structural layer of the lamina propria and submucosa. The main components of the IM are collagens I and III, fibronectin, elastin, decorin and hyaluronan (Pompili et al., 2021). The observation that Eaa binds to fibronectin, laminin, collagen I and collagen III, that are the main ECM components of the gastrointestinal tract, allows us to hypothesize that Eaa may act as an important bacterial structure to favor the adherence of aEPEC strains to the gastrointestinal tract of the host.

Fibronectin was the first ECM protein shown to act as a cellular receptor for bacterial pathogens (Kuusela, 1978). Subsequently, several other studies emphasized the importance of bacterial adherence to fibronectin as an important step to ensure host colonization, since fibronectin could act as a bridge-like structure that connects the bacteria to the host cell (Henderson et al., 2011; Izquierdo et al., 2014). Moreover, adherence of EAEC 042 to polarized T84 intestinal epithelial cells was increased by adding fibronectin. It is thought that this interaction was mediated by the aggregative adherence fimbriae II (AAF/II) (Farfan et al., 2008). The robust binding of Eaa to fibronectin may represent an additional important virulence factor for aEPEC serotype O2:H16 isolates to colonize the human gastrointestinal tract.

As already described in the literature, during catheterization of the urinary tract, fibrinogen may be released from the host, which covers the surface of the catheter (Tamadonfar et al., 2019). This fibrinogen can be used by some bacteria, such as *Enterococcus* spp., to adhere and form biofilm on the surface of the catheter, leading to persistent infections (Flores-Mireles et al., 2014). Associating the information that Eaa binds to fibrinogen and aEPEC has already been associated with symptomatic urinary tract infections (Abe et al., 2008; Nascimento et al., 2021; Tanabe et al., 2022), the binding of aEPEC to fibrinogen may favor the emergence of persistent infections in patients using urinary catheters due to this pathogen. Like Eaa, the autotransporter adhesin UpaB, identified in the uropathogenic *E. coli* CFT073 prototype, also binds to fibrinogen (Allsopp et al., 2012). Nonetheless, whether Eaa contributes to the success of urinary tract infections (UTIs) caused by hybrid aEPEC/UPEC isolates remains to be clarified, ideally through the use of *in vivo* models.

Similarly to other studies that characterized autotransporter proteins of the AIDA- I family (Torres et al., 2002; Sherlock et al., 2005; Wells et al., 2008), our study has some limitations in terms of the role of Eaa in the pathogenesis since all studies were performed *in vitro*. This limitation arises mainly from the lack of suitable animal models that allow for a more direct investigation of how different virulence factors contribute to the development of EPEC-induced diarrheal disease in the human host.

In summary, we identified a novel autotransporter adhesin in an aEPEC strain of serotype O2:H16, named EPEC autotransporter adhesin (Eaa). We characterized that this adhesin mediates bacterial autoaggregation, biofilm formation, and binding to several ECM components, especially fibronectin, which, by acting as a receptor for Eaa in the gastrointestinal tract, may contribute to the successful colonization of the host by aEPEC.

## Data Availability

The data sets presented in this study can be found in online repositories: https://repositorio.butantan.gov.br/entities/publication/91f4fbd7-4bd0-403f-955e-cf63cdf1a80a.
