# Supplementary material for: EPEC autotransporter adhesin (Eaa): a novel adhesin identified in atypical enteropathogenic Escherichia coli
Source: Front Cell Infect Microbiol. 2025 Aug 18;15:1617101. doi: 10.3389/fcimb.2025.1617101 (PMC12399667; doi:10.3389/fcimb.2025.1617101)
Supplement: Supplementary file 1 [file Table1.docx]

**Table S1.** Autotransporter proteins used in the phylogenomic analysis.

| Autotransporter protein | Protein identification |
| --- | --- |
| AIDA-I | EII49574.1 |
| Antigen 43 | YP_026164.1 |
| Calcium-binding antigen 43 homologue – Cah | AAG55356.1 |
| EHEC autotransporter encoding gene (Eha) – EhaA | AAG54657.1 |
| EhaB | AAG54720.1 |
| EhaC | AAG57362.1 |
| EhaD | AAG57760.1 |
| EhaJ | WP_137526983.1 |
| Toxigenic invasion loci A (TibA) | AAD41751.1 |
| APEC autotransporter gene (AatA) | ADJ53351.1 |
| Uropathogenic *E. coli* autotransporter (Upa) – UpaB | AAN78907.1 |
| UpaC | AAN78956.1 |
| UpaD | AAN79747.1 |
| UpaE | AAN81345.1 |
| UpaF | AAN82103.1 |
| UpaH | ACX47353 |
| UpaI | ABG70270.1 |
| EhaG | AIG71007.1 |
| UpaG | AAN82860.1 |
| UpaJ | ABG71675.1 |
| EibG | BAF32938.1 |
| STEC autoagglutinating adhesin (Saa) | AAZ76520.1 |
| STEC AT contributing to biofilm formation (Sab) | WP_011310120.1 |
| Factor adherence *E. coli* (FdeC) | ADE88959.1 |
| YeeJ | ACB16711.1 |
| Inverseautotransporter (IAT) – IatA | ACB19099.1 |
| IatB | ACB17431.1 |
| IatC | ACB17037.1 |
| IatD | ACB20062.1 |
| Intimin | WP_000627890.1 |
| Sat | VWQ04413.1 |
| SigA | WP_175055659.1 |
| Pet | SJK83553.1 |
| Pic | ALT57188.1 |
| SepA | WP_175283799.1 |
| Tsh | QEG96830.1 |
| Vat | WP_170836563.1 |
| EatA | WP_175284389.1 |
| EspP | NP_052685.1 |
| EspC | WP_133142151.1 |
