# Supplementary material for: EPEC autotransporter adhesin (Eaa): a novel adhesin identified in atypical enteropathogenic Escherichia coli
Source: Front Cell Infect Microbiol. 2025 Aug 18;15:1617101. doi: 10.3389/fcimb.2025.1617101 (PMC12399667; doi:10.3389/fcimb.2025.1617101)
Supplement: Supplementary file 2 [file Table2.docx]

**Table S2.** Gene clusters exclusively found in aEPEC isolates of serotype O2:H16.

| Gene cluster Identification | No. of aEPEC  (n = 7) | Annotation^a^ |
| --- | --- | --- |
| 196_16_11_30 | 1 (14.3%) | hypothetical protein |
| 196_16_11_31 | 1 (14.3%) | hypothetical protein |
| 196_16_1_109 | 1 (14.3%) | hypothetical protein |
| 196_16_49_2 | 6 (85.7%) | DNA (cytosine-5-)-methyltransferase family protein |
| 196_16_56_2 | 7 (100%) | phage integrase family protein |
| 282_14_12_17 | 3 (42.9%) | conserved hypothetical protein |
| 282_14_13_10 | 3 (42.9%) | hypothetical protein |
| 282_14_25_43 | 1 (14.3%) | DNA (cytosine-5-)-methyltransferase family protein |
| 282_14_25_44 | 1 (14.3%) | AAA ATPase domain protein |
| 282_14_25_77 | 6 (85.7%) | outer membrane autotransporter barrel domain protein^b^ |
| BA92_15_31 | 1 (14.3%) | conserved hypothetical protein |
| BA92_3_110 | 1 (14.3%) | putative membrane protein |
| BA92_3_111 | 1 (14.3%) | putative lipoprotein |
| BA92_3_112 | 1 (14.3%) | hypothetical protein |
| BA92_3_138 | 1 (14.3%) | conserved hypothetical protein |
| BA92_3_139 | 1 (14.3%) | phage late control gene D family protein |
| BA92_3_140 | 1 (14.3%) | hypothetical protein |
| BA92_3_141 | 1 (14.3%) | conserved hypothetical protein |
| BA92_3_143 | 1 (14.3%) | conserved hypothetical protein |
| BA92_3_144 | 1 (14.3%) | hypothetical protein |
| BA92_3_145 | 1 (14.3%) | hypothetical protein |
| BA92_3_146 | 1 (14.3%) | hypothetical protein |
| BA92_3_150 | 1 (14.3%) | hypothetical protein |
| BA92_3_151 | 1 (14.3%) | *perC* transcriptional activator family protein |
| BA92_3_152 | 1 (14.3%) | hypothetical protein |
| BA92_3_153 | 1 (14.3%) | conserved hypothetical protein |
| BA92_3_154 | 1 (14.3%) | beta family protein |
| BA92_3_155 | 1 (14.3%) | hypothetical protein |
| BA92_3_156 | 1 (14.3%) | hypothetical protein |
| BA92_3_157 | 1 (14.3%) | hypothetical protein |
| BA92_67_5 | 1 (14.3%) | conserved hypothetical protein |

^a^As previously published (Hernandes et al., 2020).

^b^This gene was renamed *eaa* (EPEC Autotransporter Adhesin) in the present study.
