# Supplementary material for: EPEC autotransporter adhesin (Eaa): a novel adhesin identified in atypical enteropathogenic Escherichia coli
Source: Front Cell Infect Microbiol. 2025 Aug 18;15:1617101. doi: 10.3389/fcimb.2025.1617101 (PMC12399667; doi:10.3389/fcimb.2025.1617101)
Supplement: Supplementary file 3 [file Table3.docx]

**Table S3.** Circular DNA molecules present in the aEPEC BA92 genome and location of the *eaa* gene.

| Circular DNA molecules present in the aEPEC BA92 strain | Length  (base pairs) | *eaa* | Accession Number |
| --- | --- | --- | --- |
| Chromosome | 4,960,033 | + | CP176406 |
| Plasmid 1 | 84,177 | - | CP176407 |
| Plasmid 2 | 43,236 | - | CP176408 |
| Plasmid 3 | 35,302 | - | CP176409 |
| Plasmid 4 | 6,647 | - | CP176410 |
| Plasmid 5 | 5,330 | - | CP176411 |
