# Supplementary material for: EPEC autotransporter adhesin (Eaa): a novel adhesin identified in atypical enteropathogenic Escherichia coli
Source: Front Cell Infect Microbiol. 2025 Aug 18;15:1617101. doi: 10.3389/fcimb.2025.1617101 (PMC12399667; doi:10.3389/fcimb.2025.1617101)
Supplement: Supplementary file 4 [file Table4.docx]

**Table S4.** Prophages identified in the chromosome of the aEPEC BA92 strain^a^.

| **Region** | **Region Lenght^b^** | **Completeness** | **Score** | **# Total Proteins** | **Region Position** | **Most Common Phage** | **GC %** | ***eaa*** |
| --- | --- | --- | --- | --- | --- | --- | --- | --- |
| 1 | 14.7 Kb | questionable | 80 | 18 | 519570-534309 | PHAGE_Salmon_118970_sal3_NC__031940(4) | 47.16% | - |
| 2 | 30 Kb | incomplete | 50 | 39 | 1499043-1529090 | PHAGE_Escher_HK75_NC_016160(9) | 45.57% | - |
| 3 | 27.3 Kb | incomplete | 50 | 41 | 1767206-1794571 | PHAGE_Entero_BP_4795_NC_004813(15) | 47.17% | - |
| 4 | 47.4 Kb | intact | 150 | 56 | 1991550-2038993 | PHAGE_Stx2_c_1717_NC_011357(9) | 45.97% | - |
| 5 | 38.1 Kb | intact | 140 | 41 | 2433101-2471221 | PHAGE_Salmon_118970_sal3_NC_031940(8) | 45.64% | - |
| 6 | 34.2 Kb | incomplete | 50 | 31 | 2649032-2683247 | PHAGE_Shigel_POCJ13_NC_025434(6) | 46.07% | - |
| 7 | 18.6 Kb | incomplete | 30 | 13 | 2912848-2931473 | PHAGE_Entero_Sfl_NC_027339(5) | 49.60% | - |
| 8 | 35.9 Kb | intact | 130 | 42 | 3075880-3111862 | PHAGE_Entero_BP_4795_NC_004813(9) | 49.33% | - |
| 9 | 22.8 Kb | questionable | 70 | 26 | 3336288-3359102 | PHAGE_Escher_SH2026Stx1_NC_049919(7) | 45.89% | - |
| 10 | 8.1 Kb | questionable | 80 | 11 | 3854854-3862978 | PHAGE_Escher_pro483_NC_028943(4) | 41.61% | + |

^a^This table was generated in the PHASTEST online tool available at https://phastest.ca/, accessed on September 3, 2024, except for the last column that was added to indicate the location of the *eaa* gene in the prophage identified as region 10.

^b^Kb: kilobase.
