# Supplementary material for: EPEC autotransporter adhesin (Eaa): a novel adhesin identified in atypical enteropathogenic Escherichia coli
Source: Front Cell Infect Microbiol. 2025 Aug 18;15:1617101. doi: 10.3389/fcimb.2025.1617101 (PMC12399667; doi:10.3389/fcimb.2025.1617101)
Supplement: Supplementary file 5 [file Table5.docx]

**Table S5.** Annotation of the genes found in a chromosomal region that harbors the *eaa* gene.

| Locus | Start position | End position | Annotation^a^ |
| --- | --- | --- | --- |
| *eaa* | 3857279 | 3859480 | EPEC Autotransporter Adhesin^b^ |
| *pinQ* | 3859611 | 3860099 | recombinase family protein |
| Locus 3 | 3860129 | 3860677 | phage tail protein |
| Locus 4 | 3860677 | 3861279 | tail fiber assembly protein |
| Locus 5 | 3861251 | 3861691 | hypothetical protein |
| Locus 6 | 3861825 | 3862118 | IS1 family transposase |
| Locus 7 | 3862247 | 3862522 | IS1 family transposase |
| Locus 8 | 3862601 | 3862978 | IS1 family transposase |
| Locus 9 | 3863050 | 3863586 | hypothetical protein |
| Locus 10 | 3863600 | 3863800 | hypothetical protein |
| Locus 11 | 3863833 | 3864045 | hypothetical protein |
| Locus 12 | 3864115 | 3865509 | hypothetical protein |
| Locus 13 | 3865512 | 3865685 | hypothetical protein |
| Locus 14 | 3865697 | 3866491 | hypothetical protein |
| *traJ* | 3866573 | 3866926 | TraJ |
| *ybaQ* | 3868196 | 3868453 | putative HTH-type transcriptional regulator YbaQ |
| Locus 17 | 3869044 | 3870408 | hypothetical protein |
| Locus 18 | 3870709 | 3871548 | hypothetical protein |
| Locus 19 | 3871538 | 3874723 | hypothetical protein |
| Locus 20 | 3875124 | 3876494 | hypothetical protein |

^a^Annotated with Prokka.

^b^Named in the present study.
