# Supplementary material for: EPEC autotransporter adhesin (Eaa): a novel adhesin identified in atypical enteropathogenic Escherichia coli
Source: Front Cell Infect Microbiol. 2025 Aug 18;15:1617101. doi: 10.3389/fcimb.2025.1617101 (PMC12399667; doi:10.3389/fcimb.2025.1617101)
Supplement: Supplementary file 6 [file SupplementaryFile1.docx]

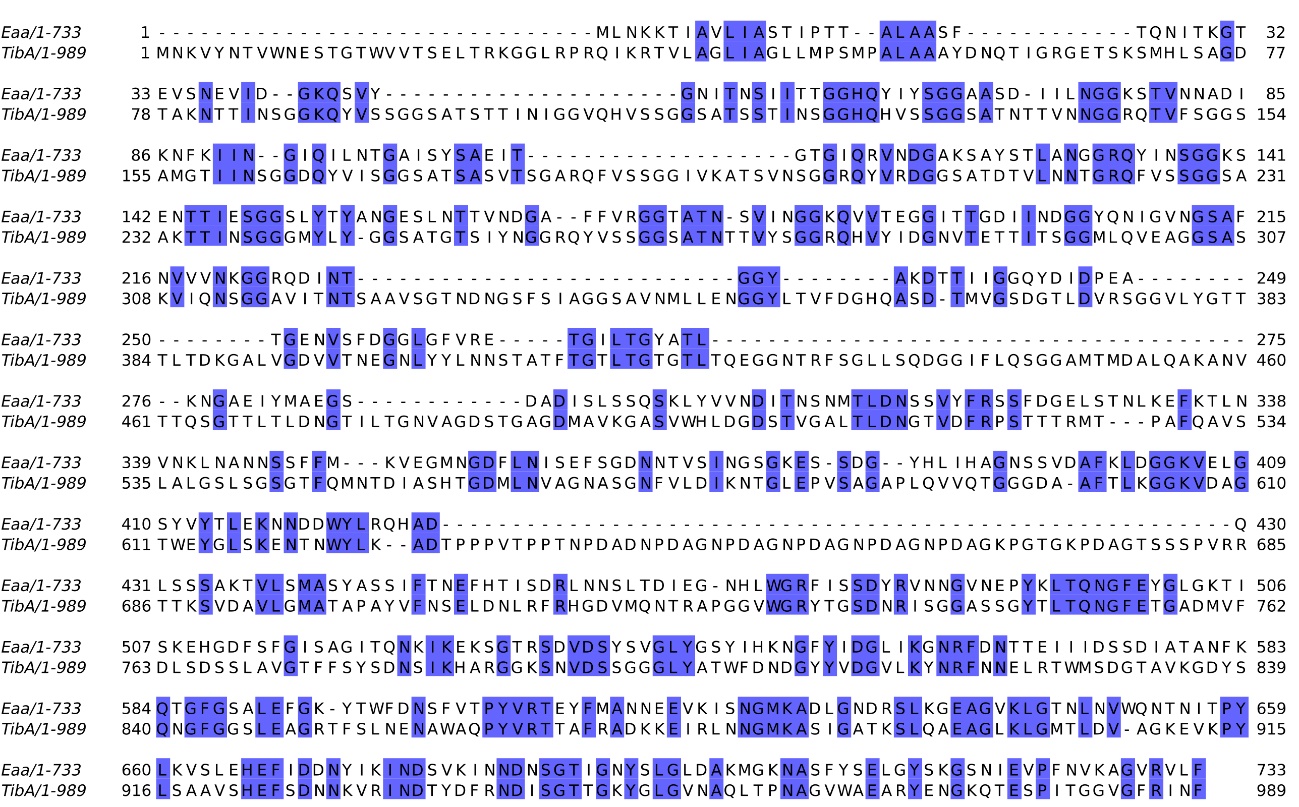


**Figure S1. Pairwise alignment of Eaa and TibA protein sequences generated by EMBOSS Needle.** Identical amino acids are highlighted with a blue background. Dashes (–) denote gaps introduced in the alignment. Overall, the alignment showed 26.2% identity (261/998), 41.1% similarity (410/998), and 27.5% gaps (274/998) between the novel autotransporter Eaa (Protein ID: XWX38401.1) and the adhesin TibA (Protein ID: AAD41751.1). Visualization of the alignment was done through Jalview 2.11.4.0.
