# Supplementary material for: EPEC autotransporter adhesin (Eaa): a novel adhesin identified in atypical enteropathogenic Escherichia coli
Source: Front Cell Infect Microbiol. 2025 Aug 18;15:1617101. doi: 10.3389/fcimb.2025.1617101 (PMC12399667; doi:10.3389/fcimb.2025.1617101)
Supplement: Supplementary file 7 [file SupplementaryFile2.docx]

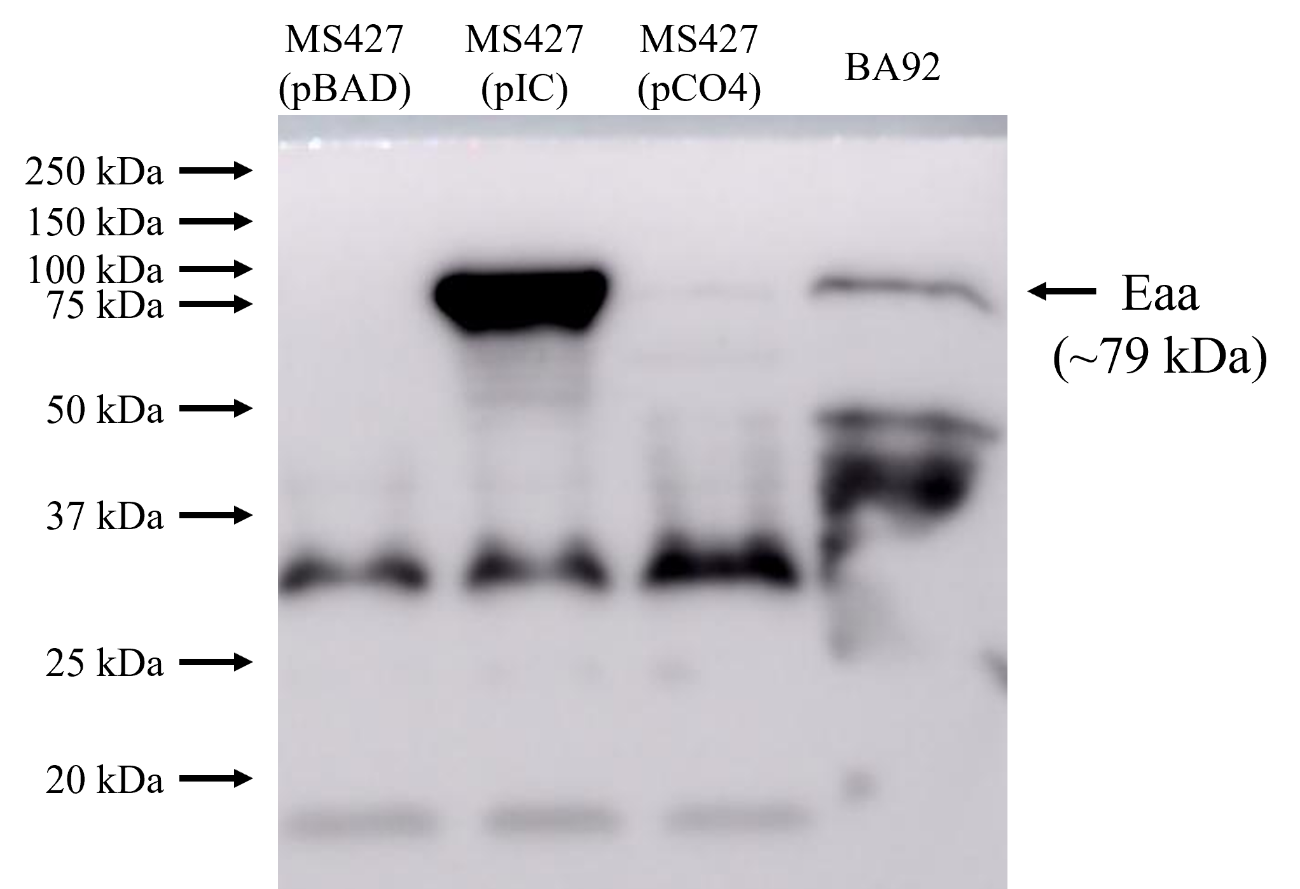


**Figure S2. Eaa is produced by the wild-type BA92 and MS427(pIC) strains.** The immunoblotting with anti-Eaa polyclonal antibody was performed using the whole cell lysates, subjected to SDS-PAGE gel electrophoresis, of the following strains: MS427(pBAD), MS427(pIC), MS427(pCO4) and the wild-type aEPEC BA92. A band of approximately 79 kDa, that corresponds to the predicted mass of Eaa, is observed in the strains MS427(pIC) and BA92, indicating that Eaa is produced by both bacteria. Molecular ladder used: Bio-Rad Precision Plus Protein Dual Color Standards.
